# Multilayer Gold-Silver Bimetallic Nanostructures to Enhance SERS Detection of Drugs

**DOI:** 10.3390/molecules25153405

**Published:** 2020-07-28

**Authors:** Marta Gambucci, Elena Cambiotti, Paola Sassi, Loredana Latterini

**Affiliations:** Dipartimento di Chimica, Biologia e Biotecnologie—Università di Perugia, via Elce di Sotto, 8, 06123 Perugia, Italy; martygam@hotmail.it (M.G.); elena.cambiotti@studenti.unipg.it (E.C.); paola.sassi@unipg.it (P.S.)

**Keywords:** gold-silver nanostructures, bi-metallic colloids, SERS, chemical enhancement, drug detection

## Abstract

Surface-enhanced Raman scattering (SERS) is a widely used technique for drug detection due to high sensitivity and molecular specificity. The applicability and selectivity of SERS in the detection of specific drug molecules can be improved by gathering information on the specific interactions occurring between the molecule and the metal surface. In this work, multilayer gold-silver bimetallic nanorods (Au@Ag@AuNRs) have been prepared and used as platforms for SERS detection of specific drugs (namely promethazine, piroxicam, furosemide and diclofenac). The analysis of SERS spectra provided accurate information on the molecular location upon binding and gave some insight into molecule-surface interactions and selectivity in drug detection through SERS.

## 1. Introduction

Surface-enhanced Raman scattering (SERS) is a non-destructive and ultra-sensitive analytical technique, which allows rapid molecular-specific detection of a wide range of targets [1,2]. Since its discovery in 1974, SERS has been applied for the analytical sensing of many molecules of biological interest ranging from small organic molecules [3,4,5], pesticides [6,7] and drugs [8,9] to larger structures such as proteins, nucleic acids and cells [10,11,12,13]. Due to its promptness, high sensitivity and molecular specificity, SERS is particularly useful for drug detection. In recent years, the technique has been applied for the detection and quantification of antibiotics [9,14] and other pharmaceutical drugs [15,16] and also for screening illicit drugs [17,18]. SERS can also provide real-time in vivo monitoring of therapeutic drugs [19,20].

The enhancement of Raman signals in SERS depends upon the intensification of the electromagnetic field of both incident and scattered radiation in the proximity of metal plasmon surfaces (electromagnetic enhancement), and/or upon the resonant or non-resonant energy transfer between the metal nanoparticle and the adsorbed molecule (chemical enhancement). Traditionally, silver-based nanomaterials are used in SERS applications, since this metal demonstrated to induce strong enhancement effects [8,9]; however, it has been already reported that silver nanostructures can easily oxidase at room temperature conditions [3] and show a strong tendency to growth and/or reshape upon prolonged irradiation [21,22]. For this reason, in recent years, the attention was focused on hybrid nanomaterials, in which the properties of two or more elements are combined in a single platform [5,7,23,24,25,26]. Hybrid materials proved to be much more effective than the separated components, showing emerging properties with respect to the sum of the properties derived from the starting elements [5,25]. Thus, SERS effect has been improved by combining different metals in alloys or core-shell structures [23,25,27] or by blending metals with metal oxides or polymers [24,26], in order to obtain multi-use nanoplatforms in which plasmonic effects are enhanced [4,23,24].

Gold and silver can be combined in hybrid bimetallic structures and the formation of core-shell structures composed by these two metals already proved to be a good strategy to improve SERS efficiency [5,25,27]. In this work, we have prepared bimetallic core-shell colloidal nanorods to be used as SERS platforms; while gold provides excellent morphological control and stability for the structure, the combination with silver strongly increases SERS efficiency. The prepared materials have been used for SERS detection of four drugs (promethazine, piroxicam, furosemide and diclofenac) used in commercially available formulations. The wide and prolonged use of these medications might rise pollution issue in urban wastewater, therefore, the chance of easily detect these molecules through SERS would be compelling. However, among the large number of commercially available drugs, these specific four molecules are chosen for their chemical properties ((molecular size, substituent groups, solubility, pKa, etc.) to explore which parameter determines a better interaction with the nanorod surface and with the stabilizer molecules (hexadecyltrimethylammonium bromide, CTAB, in our case) present on the colloids surface. The different molecular properties allow focusing the attention on the interactions with the nanostructures on a molecular level. Through the accurate analysis of the enhanced SERS bands, information about the drug disposition on the metal surface, the interactions with the metal nanostructures and with the stabilizer and the binding guiding force can be obtained. The data are fundamental to understand how molecules interact with a definite colloid and how to optimize the metal/stabilizer systems for the analytical detection of specific targets.

## 2. Results and Discussion

### 2.1. Synthesis and Characterization of Bimetallic Core-Shell Nanorods

Bimetallic silver and gold nanorods were prepared by synthesizing gold nanorods (AuNRs) through a chemical reduction method and then by growing consecutive silver (Au@AgNRs) and gold (Au@Ag@AuNRs) coating layers around the core structure. The combination of different metals in the same nanostructure allows to improve the properties of the single metals and originates a hybrid material with new enhanced characteristics, such as tunable plasmonic resonance and improved SERS efficiency [5,25]. Since gold allows an excellent size/shape control and provides high stability, this metal is an optimal candidate for the core structure and the final coating layer. On the other hand, silver has proved to induce significant SERS enhancement [8,9], therefore the addition of an intermediate silver layer improves SERS efficiency.

**Figure 1:**
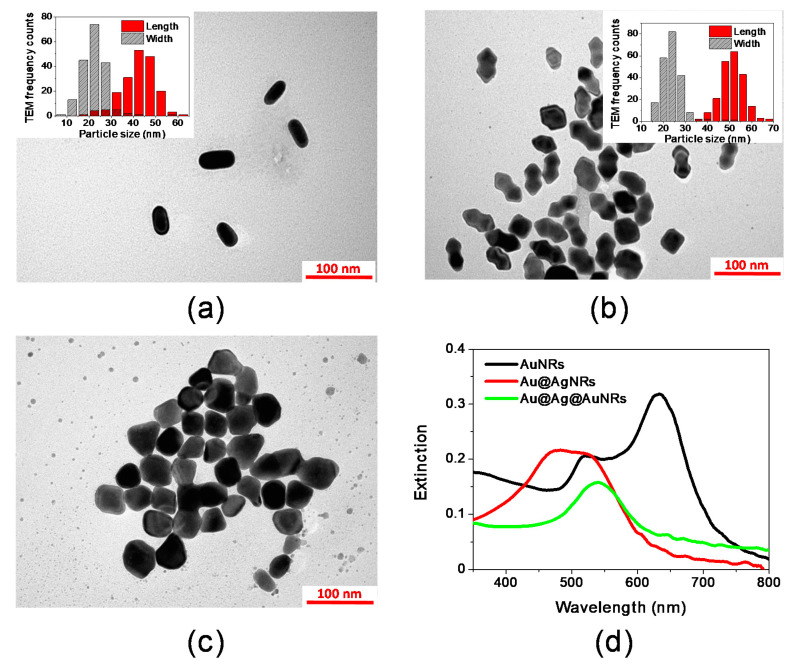
Morphological and spectroscopic characterization of AuNRs, Au@AgNRs and Au@Ag@AuNRs: (**a**) TEM image of AuNRs (scale bar 100 nm—Inset: size distribution); (**b**) TEM image of Au@AgNRs (scale bar 100 nm—Inset: size distribution); (**c**) TEM image of Au@Ag@AuNRs (scale bar 100 nm); (**d**) extinction spectra of AuNRs (black line), Au@AgNRs (red line) and Au@Ag@AuNRs (green line).

The morphological and spectroscopic properties of the synthesized AuNRs, Au@AgNRs and Au@Ag@AuNRs are presented in Figure 1 and Table 1.

Figure 1a shows a TEM representative image of AuNRs. The nanorods present a good size distribution, with an average length of 43.5 nm and an average width of 22.2 nm (Figure 1a—inset and Table 1). The two dimensions are reflected in the two plasmon bands observed in the extinction spectrum (Figure 1d, black line): the spectrum displays two maxima at 524 and 633 nm, related respectively to the transversal and longitudinal plasmons.

The coating of AuNRs with a silver layer yields Au@AgNRs. As evident in the TEM image (Figure 1b), the synthesis conditions allowed to obtain an arrow-like coating layer [4,28]. The coating slightly increases the average size of the nanorods, depositing a layer of about 3.9 nm around the AuNRs (Figure 1b—inset and Table 1). The presence of the silver layer also affects the extinction spectrum of the material; the plasmon bands shift towards lower wavelengths (480 and 522 nm) and partially overlap (Figure 1d, red line), as previously reported in the literature [28,29].

In order to improve the stability of the nanorods, a second coating was carried out by depositing a gold shell layer on Au@AgNRs surface, thus resulting in Au@Ag@AuNRs sample. It has been reported that in double shell bimetallic structures higher stability and improved SERS sensibility is achieved [30,31]. In our case however, the additional Au shell alters the morphology of the rods, inducing a partial loss of the rod-like shape: the nanorods become more irregular but they maintain pointed ends (Figure 1c). The shape irregularities of Au@Ag@AuNRs made it difficult to obtain a precise size distribution, but average length and width of about 55 and 42 nm can be respectively estimated, which correspond to a gold shell of about 3–8 nm (Table 1). The growth of the Au shell modifies the extinction spectrum of the colloids resulting in a single Surface Plasmon Resonance (SPR) band centered at 540 nm (Figure 1d, green line).

### 2.2. SERS Efficiency of Bimetallic Core-Shell Nanorods

The SERS efficiency of the prepared AuNRs, Au@AgNRs and Au@Ag@AuNRs samples is first tested on a well-known Raman reporter. These test measurements enable to verify the performances of the nanomaterials. Rhodamine 6G (Rh6G) was chosen due to its well-known efficiency as a Raman reporter. The strong N-Au (Ag) interaction and the large Raman cross section of this dye ensure a strong scattering enhancement [32] which gains further intensity by the pre-resonant conditions upon excitation at 633 nm, i.e., below the maximum absorption of the dye at about 528 nm [33]. In fact, dye detection in resonant conditions might be hindered by the fluorescence signal.

**Figure 2:**
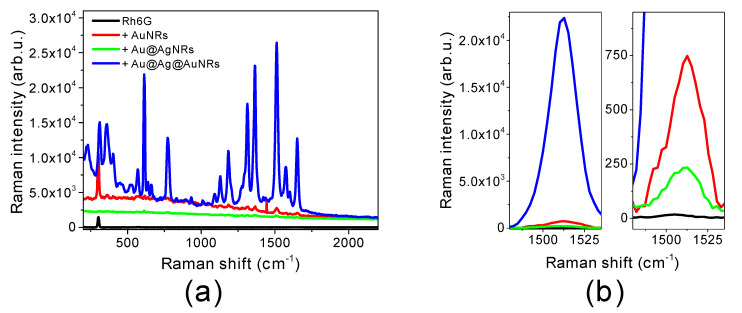
Raman spectra of 10^−5^ M Rhodamine 6G (Rh6G): (**a**) Raman spectra in aqueous solution (black line) and in the presence of AuNRs (red line), Au@AgNRs (green line) and Au@Ag@AuNRs (blue line); (**b**) magnifications of Raman peaks at 1510 cm^−1^ (spectra have been translated for a better comparison). Rh6G structure is reported as inset in graph (**a**).

Figure 2 shows Raman spectra of Rh6G (10^−5^ M) in the absence and in the presence of the three nanorod samples. The spectra show that all three types of nanomaterials induce an enhancement of Raman signals with respect to Rh6G in solution (black line), although some differences can be observed among the samples. The most intensified bands are at 357, 610, 1316, 1364 and 1510 cm^−1^; among these band, the second one (610 cm^−1^) is assigned to a xanthene ring deformation, while the others are due to a combination of vibrational modes of the xanthene ring and the two alkylated amines, mainly involving the N atoms [34,35]. The contribution of alkyl amino groups in the SERS spectra is also increased compared to literature data obtained from Rh6G crystalline samples [34], where the predominant signals are due to aromatic stretching vibrations that do not involve the amino groups (1571 and 1651 cm^−1^). These observations suggest that the alkyl amino groups are the portion of the Rh6G molecule in closer proximity with the nanorod surface and likely establish interactions through the N atoms. The interactions might be assisted by hydrogen bonding capabilities with stabilizer molecules (CTAB) and/or by the nitrogen basicity [36].

To obtain quantitative information, the intensity of Rh6G peak at 1510 cm^−1^ (assigned to a combination of xanthene ring stretching, C-N stretching and C-N-H bending [35]) was analyzed. Based on the intensities of this band, analytical enhancement factors (AEFs) for each nanorod type were calculated according to Equation (1):

(1) $$\mathrm{AEF}=\frac{I_{\mathrm{SERS}}/C_{\mathrm{SERS}}}{I_{\mathrm{Raman}}/C_{\mathrm{Raman}}}$$

where C_SERS_ and C_Raman_ are the concentrations of the probe in SERS and Raman measurements, respectively, while I_SERS_ and I_Raman_ are the corresponding SERS and Raman intensities, respectively, measured under the same experimental conditions [33]. The final AEF values are reported in Table 2 and confirm the higher efficiency of Au@Ag@AuNRs, for which AEF value is at least 200 times higher than for the other nanomaterials. It should be noted that the Rh6G concentration considered for SERS refers to Rh6G in solution and not the amount that is actually adsorbed on the nanorod surface. This last quantity could be lower than the molecule concentration in solution, therefore the determined AEFs values could be a lower limit.

The AEF values reported in Table 2 are several orders of magnitude lower than those usually observed in the presence of electromagnetic enhancement; besides, Au shows a considerably higher efficiency compared to Ag, which is generally more effective in inducing electromagnetic enhancement. These two effects suggest that, in our conditions, chemical enhancement is the prevalent type of SERS enhancement occurring. Chemical enhancement could also explain why Au@Ag@AuNRs show an excellent SERS efficiency compared to the other nanomaterials. Despite Au@AgNRs and, particularly, AuNRs presenting a higher SPR intensity at 633 nm (wavelength used as Raman source), their SERS enhancement is lower, suggesting that the local field enhancement cannot account for the determined AEF values. The establishment of chemical interactions between Au@Ag@AuNRs and portions of Rh6G molecule might have a charge transfer nature, able to affect the bond polarizability; as previously reported, nanoparticle-adsorbate charge transfer interactions boost SERS enhancement [37]. The higher performances detected for Au@Ag@AuNRs can also be due to the higher electron density on these samples’ surface. To account for the lower SERS performances of Au@AgNRs sample, photo-induced transformation of Ag upon laser irradiation, cannot be excluded.

### 2.3. SERS Aetection of Selected Drugs

Given the excellent SERS efficiency, Au@Ag@AuNRs were used for SERS detection of selected drugs. Indeed, the prevalence of a chemical enhancement mechanism in our working conditions allows a detailed characterization of molecule-surface interactions and of molecule disposition upon adsorption, providing fundamental information to improve SERS applicability in drug detection. Moreover, SERS spectrum of Au@Ag@AuNRs (Figure S1) does not show significant Raman bands, thus the nanorods can safely be used to reveal drug SERS signals without interference from the stabilizer.

**Figure 3:**
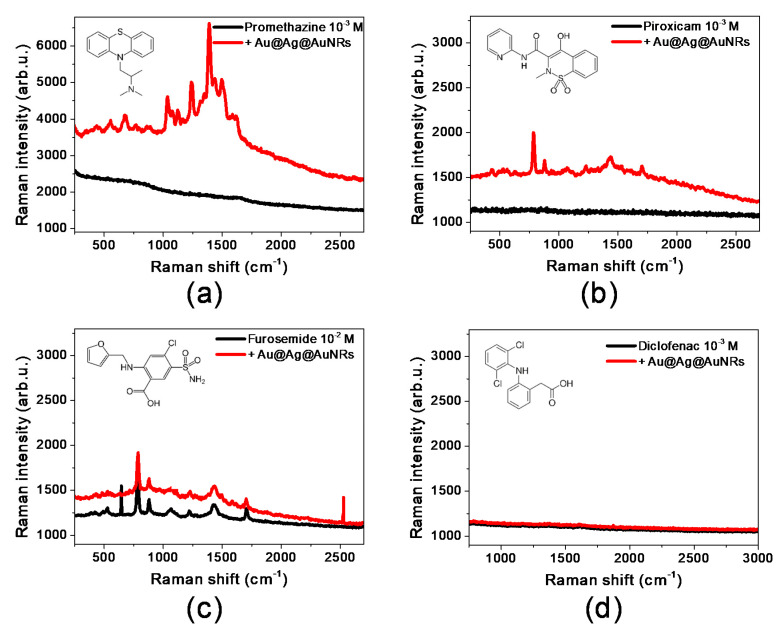
SERS enhancement of selected drug active ingredients in the presence of Au@Ag@AuNRs: (**a**) promethazine (10^−3^ M in aqueous solution); (**b**) piroxicam (10^−3^ M in ethanol solution); (**c**) furosemide (10^−2^ M in ethanol solution); (**d**) diclofenac (10^−3^ M in aqueous solution). Molecular structures of the active ingredients are reported as insets in the respective graphs.

Four drugs, present in commercially available formulations, are chosen, based on their physical and chemical properties: promethazine (antihistamine), piroxicam (NonSteroidal Anti-Inflammatory Drug, NSAID), furosemide (diuretic) and diclofenac (NSAID). The structures of the drugs are reported in Figure 3 (insets): the four molecules present common elements (aromatic portions, various heteroatoms which could bind to metal surfaces), but also several differences (charge at pH 7, heteroatom accessibility and availability for binding, hydrophobicity). In particular, promethazine is positively charged at pH 7, has a relatively high octanol/water partition coefficient (logP) compared to the other drugs (logP = 4.8) and presents N and S atoms, both of which are easily accessible. Piroxicam and furosemide are both zwitterionic at neutral pH and their logP is smaller than the one for promethazine (logP = 3.1 and 2, respectively); moreover, even if they have both N and S atoms, these atoms are less accessible with respect to promethazine. Finally, diclofenac is negatively charged at pH 7, with a logP of 4.5 and has only one N atom, which is fairly hindered by the aromatic portions of the molecule.

Figure 3 shows the Raman spectra of the selected drug molecules in the absence and in the presence of Au@Ag@AuNRs. The best SERS enhancement is obtained for promethazine, whose bands in the presence of Au@Ag@AuNRs are 3.5 times higher than in aqueous solution (Figure 3a and Table 3). The best-enhanced bands in SERS spectrum are at 1494, 1389 and 1238 cm^−1^: as reported elsewhere [38], these bands are respectively assigned to CH_3_ deformation, NH bending and CH_3_ rocking, suggesting that the molecule interacts with the metal surface through the terminal N atom. The hypothesis is confirmed by the fact that Raman bands related to the aromatic portion of the molecule (for example C-C stretching modes at 1570 and 1034 cm^−1^ [38]) are barely visible in the SERS spectrum, although they are predominant in the Raman spectrum of promethazine powder (Figure S2).

Piroxicam and furosemide both show a modest increase in Raman intensity in the presence of Au@Ag@AuNRs (Figure 3b,c and Table 3). The most intense band in piroxicam SERS spectrum (786 cm^−1^) corresponds to bending modes of the aromatic rings [39]. Other relevant bands are present at 878 cm^−1^ (another aromatic bending mode [39]) and 1438 cm^−1^ (assigned to CH_3_ bending on the N atom of the thiazinic ring [39]), indicating that the molecule is most likely bound to the metal surface through the thiazinic ring. Bands related to SO_2_ stretching (1279 and 1152 cm^−1^ for the solid sample, Figure S3) are hardly visible in the SERS spectrum, suggesting that this group is barely involved in the binding, which therefore occurs through the thiazinic N atom.

Furosemide SERS spectrum shows again relatively intense band at 786 cm^−1^ (assigned to aromatic bending modes, with some contribution of COO^−^ group [40]), together with additional bands at 880 and 1435 cm^−1^. While the first one corresponds to an aromatic bending mode, the second one can be assigned to CH_2_ bending in the aliphatic chain [40]. Also in this case, the absence of SO_2_ stretching and NH_2_ bending bands (both present in furosemide powder spectrum, respectively at 1148 and 1072 cm^−1^, Figure S4) suggests that the binding occurs through the alkyl-amino group.

Finally, diclofenac shows no detectable Raman bands in solution and no SERS enhancement in the presence of the nanorods (Figure 3d); however, characteristic bands such as aromatic stretching modes at 1600 cm^−1^, the aromatic bending mode at 1045 cm^−1^ and the CN stretching at 1234 cm^−1^ [41] can be observed for the solid sample (Figure S5). It is therefore reasonable to assume that the molecule cannot effectively bind to the metal surface, probably due to steric hindrance of the N atom.

The collected data prove that drug adsorption and binding on CTAB-stabilized Au@Ag@AuNRs surface is not related to the presence of aromatic portions in the molecular structure (since this portion is only slightly involved in the binding) or molecule hydrophobicity (since promethazine and diclofenac show very similar LogP values but totally different SERS response). On the other hand, molecular charge seems to play a role in directing the interaction, because molecules presenting positive charges show a higher SERS enhancement and therefore a stronger affinity for the nanorods. The main driving force for the interaction, however, is the presence and accessibility of N atoms; accessibility of binding site ensures the reduced distance (<5 nm) between the drug and the metal surface that is necessary to obtain a SERS effect. When the N atom is easily approachable (such as for promethazine), the binding is favored and a strong SERS enhancement can be obtained as a result. On the other hand, by progressively diminishing the accessibility of the N atom, the affinity towards the surface decreases and lower or no SERS enhancement can be observed.

### 2.4. Concentration Effects on SERS Signals

**Figure 4:**
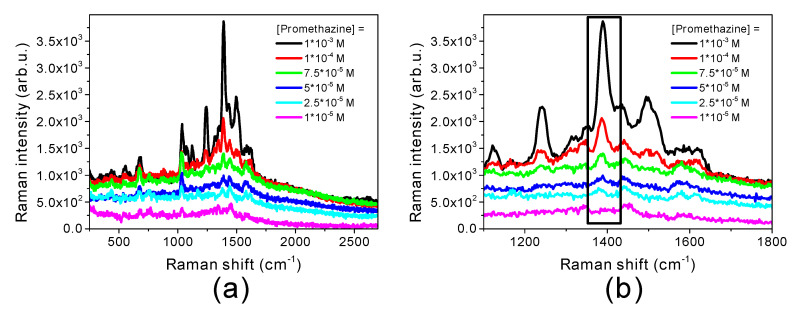
SERS detection limit for promethazine in the presence of Au@Ag@AuNRs: (**a**) Raman spectra of promethazine at different concentrations; (**b**) magnification of promethazine Raman signal at 1389 cm^−1^.

Since promethazine showed the best SERS enhancement in the presence of Au@Ag@AuNRs, the concentration of the drug was gradually lowered to evaluate the concentration effects on the SERS spectrum. Figure 4a presents Raman spectra of promethazine with concentrations ranging from 10^−3^ M to 10^−5^ M. The same amount of Au@Ag@AuNRs was used in all the samples and the Raman spectrum of Au@Ag@AuNRs is subtracted to each promethazine spectrum in order to eliminate every non-drug related contribution. The most intense promethazine Raman band (1389 cm^−1^, assigned to CNH bending [38]) is the one that is detectable even at reduced concentrations (Figure 4b). The obtained results prove that, through SERS enhancement, it is possible to reveal promethazine concentrations as low as 10^−5^ M. Below this concentration, Raman signals of the drug are not distinguished from the noise under the experimental conditions here used.

## 3. Materials and Methods

### 3.1. Materials

Gold (III) chloride trihydrate (HAuCl_4_·3H_2_O, +99.9%), hexadecyltrimethylammonium bromide (CTAB, ≥98%), sodium borohydride (NaBH_4_, 98%), silver nitrate (AgNO_3_, 99%), L-ascorbic acid (AA, reagent grade), glycine (>99%) and Rhodamine 6G (Rh6G, 95%) were all purchased from Sigma Aldrich. Promethazine hydrochloride (Ph.Eur.), piroxicam (Ph.Eur.) and furosemide (Ph.Eur.) were purchased from A.C.E.F. s.p.a. (Fiorenzuola D’Arda, Piacenza, Italy), while diclofenac (Ph.Eur.) was purchased from Sigma Aldrich. All aqueous solutions were prepared with Milli-Q water. Ethanol (EtOH, 96%) was purchased from Sigma Aldrich.

### 3.2. Synthesis of AuNRs

CTAB-stabilized gold nanorods (AuNRs) have been synthesized through the seed-mediated growth reported by El-Sayed [42,43]. At first, a 0.5 mM HAuCl_4_·3H_2_O solution in 0.2 M CTAB was put under stirring at 27 °C. A cold 10 mM NaBH_4_ solution was then added to a final ratio of [NaBH_4_]:[HAuCl_4_] = 2:1, resulting in the formation of Au spherical seeds. In a second step, a growth solution 1 mM in HAuCl_4_ and 4 mM in AgNO_3_ was prepared in 0.2 M CTAB and kept under stirring at 27 °C. To this solution, 0.078 ML-ascorbic acid was added to a final ratio of [AA]:[HAuCl_4_] = 1:1. After the discoloration of the solution due to reduction of Au(III) to Au(I), the previously prepared Au seeds were added and the solution was left under stirring at 27 °C for 2 h. The slow color change to deep blue indicated the formation of AuNRs. The obtained CTAB-AuNRs were then washed once with Milli-Q water by centrifugation, to remove the excess of CTAB and any residue of the reagents (sample AuNRs).

### 3.3. Synthesis of Bimetallic Core-Shell Au@AgNRs and Au@Ag@AuNRs

The coating of AuNRs with a silver layer was carried out according to the procedure reported by Bai et al. [28]. Briefly, the AuNRs obtained as described in the previous section were dispersed in 5 mL of CTAB 0.1 M and then 5 mL of glycine 0.2 M and 30 µL of NaOH 2 M were added. The solution was kept at 29 °C under stirring for 10 min, after which 40 µL of HAuCl_4_ 25.4 mM, 0.2 mL of AgNO_3_ 10 mM and 0.2 mL of AA 0.1 M were added. The reaction mixture was kept under stirring at 29 °C for 2 h and then the obtained CTAB-Au@AgNRs were washed once with Milli-Q water by centrifugation (sample Au@AgNRs).

The same protocol was used for the second coating, only this time a larger amount of gold and a smaller amount of silver were added. The obtained Au@AgNRs were dispersed in 5 mL of CTAB 0.1 M, after which 5 mL of glycine 0.2 M and 30 µL of NaOH 2 M were added. The solution was stirred at 29 °C for 10 min and then 250 µL of HAuCl_4_ 10 mM, 40 µL of AgNO_3_ 10 mM and 275 µL of AA 0.01 M were added. After 2 h under stirring at 29 °C, the obtained CTAB-Au@Ag@AuNRs were washed once with Milli-Q water by centrifugation (sample Au@Ag@AuNRs).

### 3.4. Characterization of Nanomaterials

A Philips (Amsterdam, The Nederlands) transmission electron microscope (mod. 208, operating at 80 kV of beam acceleration) was used to analyze the nanorod size distribution. The nanorod suspensions were deposited on a 300-mesh carbon film coated copper support grid and were left overnight in a desiccator to allow the solvent to evaporate. The size distribution histograms for the samples was obtained by analyzing TEM images through Image J software, counting at least 150–200 nanoparticles. The experimental size histograms were then reproduced using the Gaussian function to obtain the dispersion of particle dimension, evaluated by the standard deviation (σ) parameter. Extinction spectra were recorded on a Cary 8454 UV-VIS Diode Array spectrophotometer, using a 1 cm path length quartz cuvette.

### 3.5. Raman Measurements

For Raman measurements, each nanorod sample was precipitated and redispersed either in a 10^−5^ M aqueous solution of Rhodamine 6G (Rh6G) or in a solution of promethazine (10^−3^ M in water), piroxicam (10^−3^ M in EtOH), furosemide (10^−2^ M in EtOH) or diclofenac (10^−3^ M in water).

Raman spectra were collected using a micro-Raman setup equipped with a He-Ne laser (Melles-Griot, Rochester, NY United States mod. 25LHP925) emitting at λ = 632.8 nm whose power on the sample was kept at 5 mW, ca. Samples were prepared by depositing each suspension on a germanium substrate. A back-scattering geometry was realized using the 50× long working distance objective of an OLYMPUS (Shinjuku, Tokyo, Japan) microscope MOD BX40, equipped with a digital camera. The scattered radiation was analyzed by an iHR320 imaging spectrometer Horiba Jobin-Yvon (Kyoto, Japan). The signal was dispersed by a 600 grooves/mm grating which allowed spectra acquisition in the 63–2691 cm^−1^ range and spectra were recorded as an average of 10 scans, each one accumulated within 30 s or 60 s integration time (depending on the sample) at 8 cm^−1^ resolution.

## 4. Conclusions

Multilayer gold-silver bimetallic nanorods have been prepared as platforms for SERS detection of selected drug molecules, focusing on understanding how the single molecules bind on the metal surface. The combination of two different metals for the synthesis of the nanorods combined the properties of both gold and silver, thus obtaining an enhanced platform with better stability and higher SERS activity. The synthesized Au@Ag@AuNRs produce a SERS enhancement that is prevalently (if not exclusively) chemical: as a result, detailed information about the location of adsorbed analytes can be obtained.

Four commercially available drugs (namely promethazine, piroxicam, furosemide and diclofenac) were selected as analytes for SERS detection with Au@Ag@AuNRs. SERS spectra showed that the best enhancement is obtained for promethazine. Only a moderate effect is observed for piroxicam and furosemide, while no Raman signals could be detected in the case of diclofenac at millimolar concentration. A detailed analysis of the SERS enhanced bands for each molecule allowed to establish that the drug molecule preferentially bind to the metal surface through N atoms and that the binding occurs (and leads to an efficient SERS enhancement) only when N atoms are easily accessible.

Finally, the effects of promethazine concentration on its SERS signal is evaluated, revealing promethazine concentrations as low as 10^−5^ M at 5 mW of 633 nm excitation.

## Figures and Tables

**Table 1:** Average lengths, widths and aspect ratios obtained from TEM analysis of AuNRs, Au@AgNRs and Au@Ag@AuNRs.

Sample	Length (nm)	Width (nm)	Aspect Ratio	Δ_lenght_ (nm)
AuNRs	43.5 (σ = 6.4)	22.2 (σ = 4.7)	2.00 (σ = 0.30)	---
Au@AgNRs	51.2 (σ = 4.9)	23.3 (σ = 4.0)	2.22 (σ = 0.48)	7.7
Au@Ag@AuNRs	≈55	≈42	≈1.3	≈2.8

**Table 2:** Analytical enhancement factors (calculated based on Raman peak at 1510 cm^−1^) for Rh6G in the presence of AuNRs, Au@AgNRs and Au@Ag@AuNRs.

Sample	AEF
Rh6G	---
Rh6G + AuNRs	18 ± 2
Rh6G + Au@AgNRs	4.9 ± 0.5
Rh6G + Au@Ag@AuNRs	3650 ± 370

**Table 3:** Raman shift and intensity of reference signals for promethazine, piroxicam, furosemide and diclofenac in aqueous solution and in the presence of Au@Ag@AuNRs.

Sample	Raman Shift (cm^−1^)	Raman Intensity of Reference Signal
Promethazine	1389	55
+ Au@Ag@AuNRs		3170
Piroxicam	786	32
+ Au@Ag@AuNRs		441
Furosemide	786	294
+ Au@Ag@AuNRs		416
Diclofenac	[Not detectable]	[Not detectable]
+ Au@Ag@AuNRs		[Not detectable]

## Supplementary Materials

The following are available online, Figure S1: Raman spectrum of Au@Ag@AuNRs; Figure S2: Raman spectrum of promethazine powder; Figure S3: Raman spectrum of piroxicam powder; Figure S4: Raman spectrum of furosemide powder; Figure S5: Raman spectrum of diclofenac powder.
