# Supplementary material for: Multilayer Gold-Silver Bimetallic Nanostructures to Enhance SERS Detection of Drugs
Source: Molecules. 2020 Jul 28;25(15):3405. doi: 10.3390/molecules25153405 (PMC7436262; doi:10.3390/molecules25153405)
Supplement: Supplementary file 1 [file molecules-25-03405-s001.pdf]

Supporting Information

Multilayer Gold-Silver Bimetallic nanostructures to enhance SERS detection of drugs

Marta Gambucci, Elena Cambiotti, Paola Sassi and Loredana Latterini *

Dipartimento di Chimica, Biologia e Biotecnologie - Università di Perugia, via Elce di Sotto, 8, 06123 Perugia, Italy
* Correspondence: loredana.latterini@unipg.it; Tel.: +39-75-585-5583; Fax: +39-75-585-5598 (L.L.)

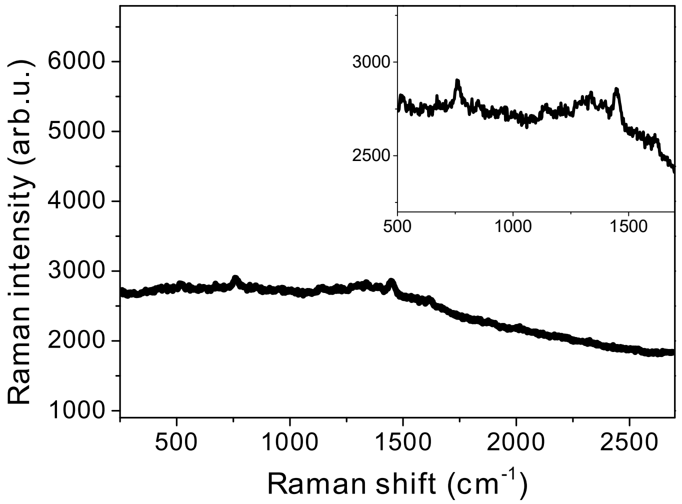

Figure S1: Raman spectrum of Au@Ag@AuNRs, recorded in the same conditions used for subsequent SERS measurements on drugs. Inset: magnification.

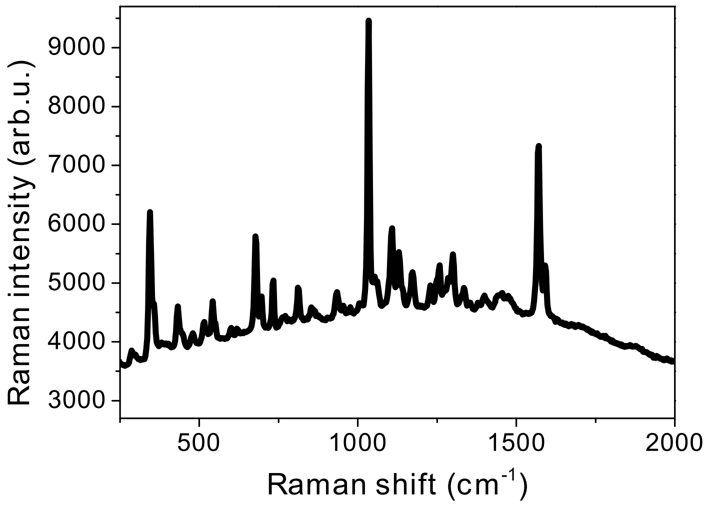

Figure S2: Raman spectrum of promethazine powder.

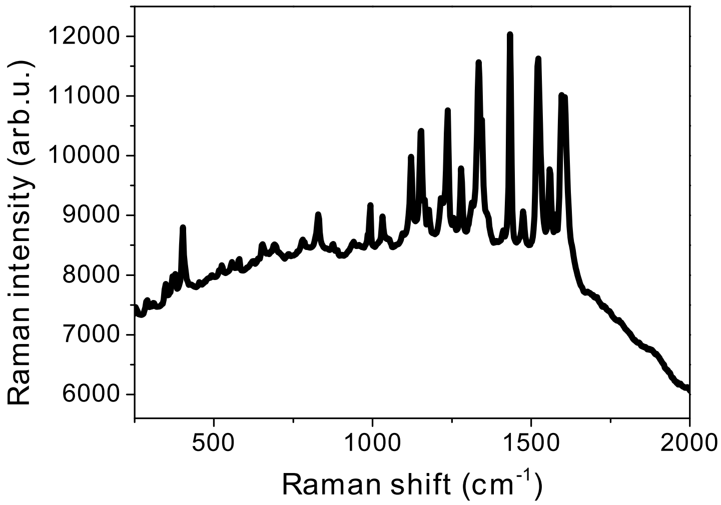

Figure S3: Raman spectrum of piroxicam powder.

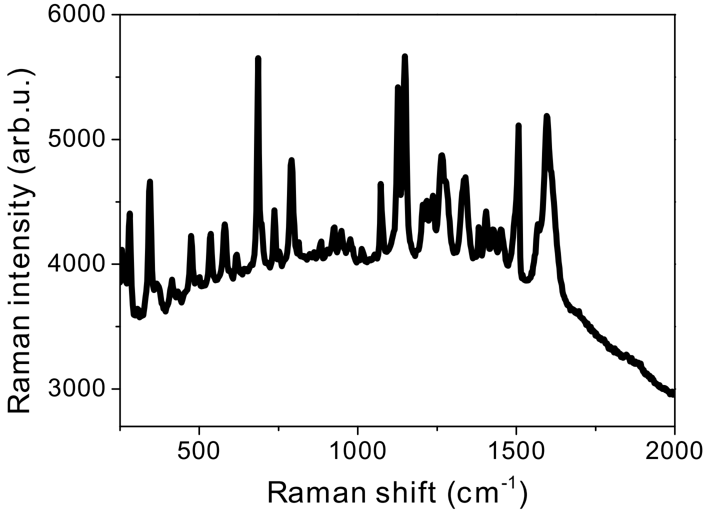

Figure S4: Raman spectrum of furosemide powder.

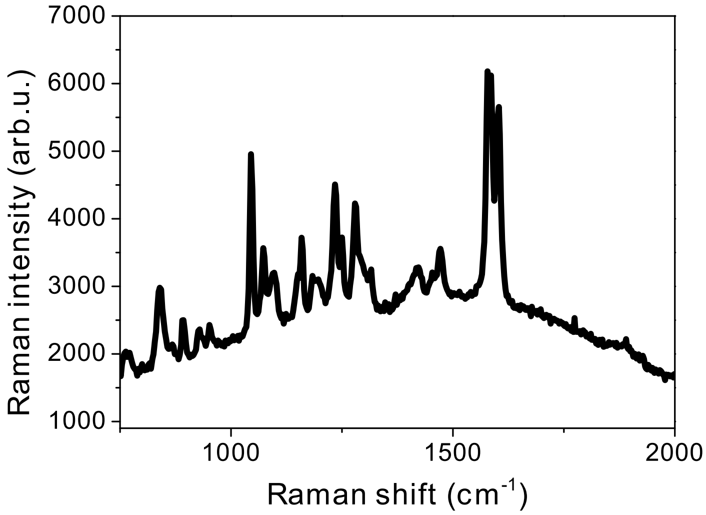

Figure S5: Raman spectrum of diclofenac powder.
